# Emphysematous pyelonephritis in type II diabetes: A case report of an undiagnosed ureteric colic

**DOI:** 10.1186/1757-1626-1-192

**Published:** 2008-09-30

**Authors:** Samuel R Vollans, Ranjit Sehjal, James A Forster, Karol M Rogawski

**Affiliations:** 1 Department of Urology, Huddersfield Royal Infirmary, Acre Street, Lindley, Huddersfield, HD3 3EA, UK

## Abstract

**Introduction:**

Emphysematous pyelonephritis (EPN) is a severe acute necrotising infection of the renal parenchyma and perirenal tissue, characterised by gas formation. 90% of cases are seen in association with diabetes mellitus. We report a case of undiagnosed ureteric obstruction in a type II diabetic, leading to EPN requiring emergency nephrectomy.

**Case presentation:**

A 59-year-old type II tablet controlled diabetic woman presented complaining of a five day history of right sided abdominal pain associated with vomiting, abdominal distension and absolute constipation. There were no lower urinary tract symptoms. Past surgical history included an open appendectomy and an abdominal hysterectomy. On examination, she was haemodynamically stable, the abdomen was soft, distended, and tender in the right upper and lower quadrants with no bowel sounds. Investigations revealed a CRP of 365 and 2+ blood and nitrite positive on the urine dipstick. The AXR was reported as normal on admission, however when reviewed in retrospect revealed the diagnosis. She was managed, therefore, as having adhesional bowel obstruction and a simple UTI. After four days, a CT was organised as she was not settling. This showed a right pyohydronephrosis with gas in the collecting system secondary to an 8 mm obstructing ureteric calculus. The kidney was drained percutaneously via a nephrostomy and the patient was commenced on a broad spectrum intravenous antibiotics. Despite this, she went on to need an emergency nephrectomy for uncontrolled severe sepsis. She was discharged in good health 15 days later.

**Conclusion:**

EPN carries a mortality of up to 40% with medical management alone. Early recognition of EPN in an obstructed kidney is essential to guide aggressive management, and in the presence of continued severe sepsis or organ dysfunction an urgent nephrectomy should be carried out. Diabetic patients who are known to have renal or ureteric calculi, whether symptomatic or not, should be considered for percutanous or ureteroscopic treatment. In the acute abdomen, the plain abdominal radiograph should always be viewed with respect to general surgical, vascular and urological differential diagnoses.

## Introduction

Emphysematous pyelonephritis (EPN) is a severe acute necrotising infection of the renal parenchyma and perirenal tissue, characterised by gas formation. The condition presents amongst other things with abdominal pain, septic shock, vomiting, fever, lethargy and confusion. The majority of cases reported are unilateral, occur in patients with diabetes mellitus or urinary tract obstruction, and more commonly affect on the left kidney [1,2]. We report a case of 'missed' ureteric obstruction in a type II diabetic, leading to EPN requiring an emergency nephrectomy.

## Case presentation

A 59-year-old white Caucasian woman presented to the Emergency Department complaining of a five day history of right sided abdominal pain, which had got progressively worse over the preceding few hours. The pain was limited to the right side of the abdomen with no radiation, and was described as an ache. Associated symptoms included nausea, vomiting and abdominal distension. Her bowels had not been open for three days, and she had not passed flatus for the last twenty-four hours. She did not complain of any lower urinary tract or gynaecological symptoms.

Past surgical history included an open appendectomy 15 months previously and an abdominal hysterectomy. Past medical history included tablet controlled (metformin and gliclizide) type II diabetes mellitus.

On admission she was alert, orientated, apyrexial and haemodynamically stable. Examination of the cardiovascular system was unremarkeable, and the only abnormality on respiratory examination was reduced breath sounds at the right lower zone. The patient's abdomen was distended but soft, and on palpation she complained of severe pain in the right upper and lower quadrants with no guarding. Bowel sounds were absent, and PR examination was normal. FBC, U&E, CRP, Glucose, CXR, AXR and urine dipstick were ordered.

The FBC and U&E were normal (Hb12.6 g/dl, WCC11.0 × 10^9^/l, Ur 4 mmol/l, Cr 86 μmol/l), serum glucose was elevated at 12.7 mmol/L, and the CRP was grossly elevated (365 mg/l). The erect CXR was normal with no free gas under the diaphragm. A plain AXR was reported as showing gas in the transverse colon and rectum. Urine dipstick showed 2+ blood and was positive for nitrites. A sample was sent for microbiology and the patient was prescribed trimethoprim. At this stage the diagnosis of adhesional bowel obstruction was made, as well as an incidental UTI.

Though noting that the ileus was somewhat unusual, the surgical team concurred with the diagnosis, as she had been admitted with similar abdominal pains in the past under the general surgeons which had settled spontaneously. She was made "nil-by-mouth", intravenous fluids were commenced and a nasogastric tube was inserted for her persistent vomiting of bilious fluid.

Urine culture grew *Escherichia coli*, sensitive to Trimethoprim which was continued. Blood WCC had increased to 14.8 × 10^9^/l by Day 2, and renal function remained normal. On Day 3, a repeat urine culture was sent and this result came back on Day 4, when the patient had begun to deteriorate significantly; it revealed a growth of *Escherichia coli*, now resistant to trimethoprim, and an *Enterococcus sp*. resistant to cefuroxime. Both were sensitive to ciprofloxacin. She was thus commenced on intravenous Ciprofloxacin, Flucloxacillin and Metronidazole, and a CT scan of the abdomen was organised.

**Figure 1:**
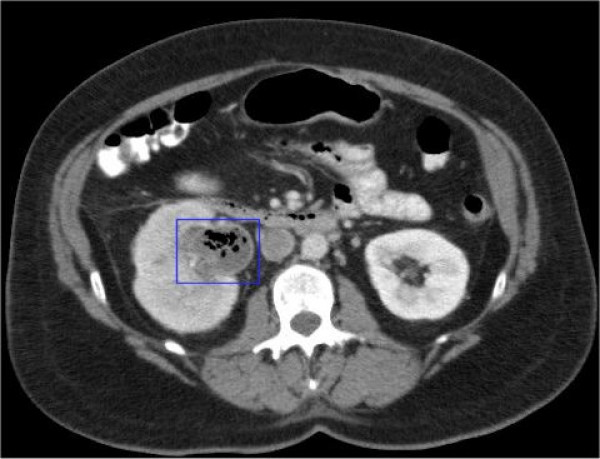
CT scan showing a moderate hydronephrosis, with gas in a dilated pelvicalyceal system.

**Figure 2:**
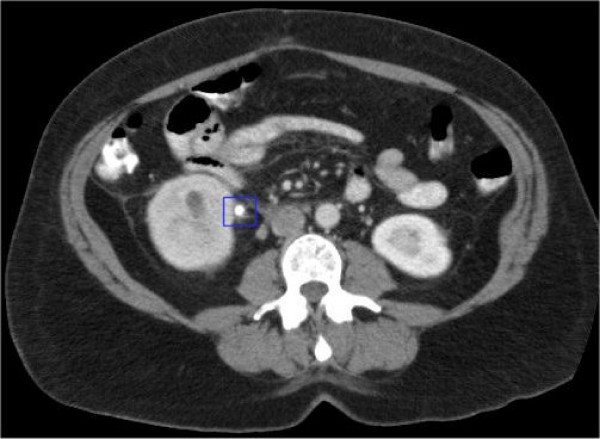
CT scan showing an 8 mm calculi at the right PUJ.

The CT scan revealed a moderate hydronephrosis, with gas in a dilated pelvicalyceal system (Figure 1) due to an 8 mm stone at the right pelvi-ureteric junction (PUJ) (Figure 2). The right ureter was completely collapsed distal to the stone. Prompt urological input was sought, which lead to an immediate USS guided right nephrostomy with a 6-French catheter and frank pus was drained. Over the next 24 hours, the patient's condition deteriorated further, showing signs of severe sepsis with a compensated metabolic acidosis (pH 7.38, CO_2 _3.3 kPa, HCO_3_^- ^18 mmol/l, Base Excess -10 mmol/l, Lactate 8.6 mmol/l). In addition, the percutaneous nephrostomy was draining little urine, and thus an emergency right nephrectomy via a loin incision was performed after resuscitation and stabilization. Extraperitoneal dissection and excision of the kidney was technically challenging due to the acute timing of surgery, but proceeded without complication.

Following the operation the patient was admitted to the Intensive Care Unit for invasive monitoring, cardiovascular and respiratory support, and blood glucose control. She was extubated successfully on post-operative day 3 and returned to the ward soon after for recovery, leading to discharge, 15 days after admission. When reviewed in out-patients' clinic, she had no complaints, was free of urinary infection and had normal renal function.

**Figure 3:**
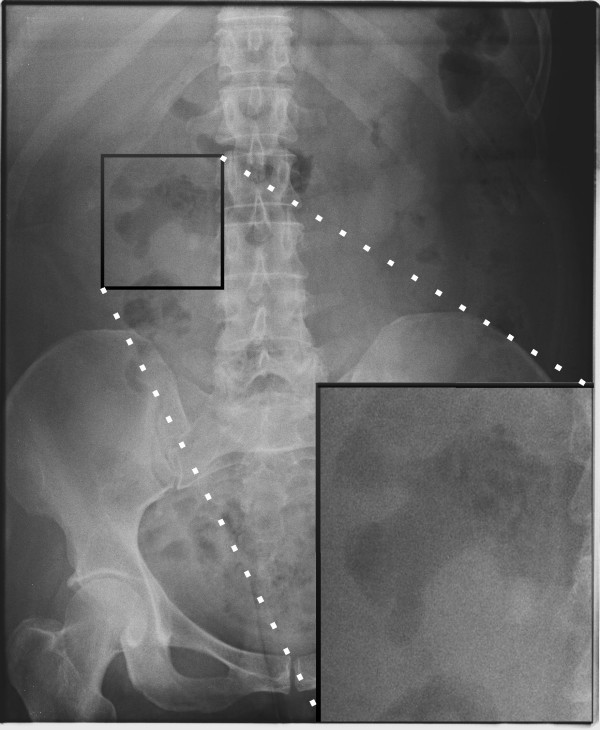
Plain AXR showing a gas filled right renal pelvis with a visible calcification, consistent with a right EPN secondary to a proximal ureteric stone.

The case was reviewed at the urology x-ray meeting during her recovery on the ward. In retrospect, the admission AXR showed a large calculus at the level of the L3 vertebra on the right, and gas in the right renal pelvis (Figure 3). A CT scan performed four months previously to investigate a change in bowel habit and right-sided abdominal pain, demonstrated a 7.4 mm calculus within the renal pelvis on the right side.

## Discussion

EPN was first described in 1898, in association with pneumaturia as a result of gas forming pathogens [3]. The most common pathogen is *Escherichia Coli *(70%), followed by *Klebsiella pneumoniae *(29%) and *Proteus *[1]. These bacteria ferment sugars within the urine producing gases including nitrogen, hydrogen, carbon dioxide, and oxygen [4]. EPN occurs nearly exclusively (90%) in people with diabetes [1]. The exact pathophysiology of EPN is still unclear. This is evidenced on the observation that UTIs are very common in diabetic patients, and only a small proportion of these patients develop EPN. The factors that predispose to EPN in people with diabetes may include uncontrolled diabetes, high levels of glycosylated hemoglobin, and impaired host immune mechanisms caused by local factors such as renal tract obstruction (tumours or lithiasis).

With regard to imaging the AXR, although reported as normal, demonstrated gas in the collecting system of the right kidney and a calculus at the PUJ. In the acute abdomen, the AXR should specifically be reviewed to exclude signs of all general surgical diagnoses, as well as vascular (aneurysms) and urological differential diagnoses.

Renal USS can confirm the presence of EPN in approximately 80% of cases [5], whereas CT is 100% sensitive [6]. Thus, a CT scan is mandatory to diagnose EPN if the index of suspicion is high. The most recent CT classification of EPN is described by Huang et al [1], with minor adjustments from the previously proposed classification by Michaeli et al (1984) [7]. It essentially describes the anatomical location of gas on CT scan:

• **Class 1 **– Gas confined to the collecting system

• **Class 2 **– Gas confined to the renal parenchyma alone

• **Class 3a **– Perinephric extension of gas or abscess

• **Class 3b **– Extension of gas beyond the Gerota fascia

• **Class 4 **– Bilateral EPN or unilateral EPN with a solitary kidney

It is now largely accepted that nephrectomy is the treatment of choice in most patients with EPN [7]. When treated with antibiotics alone, EPN is associated with a high mortality rate (40%) [8]. Huang *et al *concluded that Class 1 and Class 2 EPN *could *be managed with percutaneous drainage and antibiotics [1]. In class 3 and class 4 EPN, the presence of fewer than two risk factors (thrombocytopenia, acute renal failure, stupor/coma and shock) indicated that percutaneous drainage and antibiotics *could *also be used (successful in less than 64% of cases). However, in the presence of three or more of the above risk factors, nephrectomy yielded better results. Mortality rates were 15–20% in two other case series in which nephrectomy was the treatment of choice [2,9].

Our patient had Class 1 EPN, and underwent an emergency nephrectomy following persistent deterioration despite percutaneous nephrostomy and adequate intravenous antibiotics. Thus, the authors of this case concur with others in suggesting that early nephrectomy is recommended in any severe presentation of EPN (*i.e. *with septic shock, severe sepsis or multi-organ dysfunction syndrome) [1,10].

## Conclusion

EPN is a condition which carries major morbidity and significant mortality. Rapid and thorough assessment, prompt diagnosis and appropriate aggressive treatment is likely to reduce mortality in these life-threatening cases of urinary tract obstruction in diabetics. As 90% of EPN occurs in patients suffering from diabetes mellitus, one should consider early intervention of renal and ureteric calculi, whether symptomatic or not, to prevent potentially devastating complications of EPN.

## Abbreviations

CRP: C-reactive protein; AXR: abdominal x-ray; UTI: urinary tract infection; CT: computed tomography; FBC: full blood count; U&E: urea & electrolytes; CXR: chest x-ray; Hb: Haemoglobin; WCC: white cell count; Ur: urea; Cr: creatinine; USS: ultrasound scan.

## Competing interests

The authors declare that they have no competing interests.

## Authors' contributions

SRV and KMR were involved in the medical management of the patient. KMR undertook the surgical interventions on the patient. SRV obtained written consent from the patient for publication of information and images regarding the case. SRV and RS carried out the literature search and produced the draft manuscript. JAF reviewed and revised the draft manuscript. All authors reviewed and approved the final manuscript.

## Consent

Written informed consent was obtained from the patient for publication of this case report and accompanying images. A copy of the written consent is available for review by the Editor-in-Chief of this journal.

## References

[B1] Huang JJ, Tseng CC (2000). Emphysematous pyelonephritis: Clinicoradiological classification, management, prognosis and pathogenesis. Arch Intern Med.

[B2] Shokeir AA, El-Azab M, Mohsen T, El-Diasty T (1997). Emphysematous pyelonephritis: a 15-year experience with 20 cases. Urology.

[B3] Kelly HA, MacCallum WG (1898). Pneumaturia. JAMA.

[B4] Huang JJ, Chen KW, Ruaan MK (1991). Mixed acid fermentation of glucose as a mechanism of emphysematous urinary tract infection. J Urol.

[B5] Tang HJ, Li CM, Yen MY, Chen YS, Wann SR, Lin HH (2001). Clinical characteristic of emphysematous pyelonephritis. J Microbiol Immunol Infect.

[B6] Ahlering TC, Boyd SD, Hamilton CL (1985). Emphysematous pyelonephritis: a five year experience with 13 patients. J Urol.

[B7] Michaeli J, Mogle P, Perlberg S, Heiman S, Caine M (1984). Emphysematous pyelonephritis. J Urol.

[B8] Wan YL, Lo SK, Bullard MJ, Chang PL, Lee TY (1998). Predictors of outcome in emphysematous pyelonephritis. J Urol.

[B9] Pontin AR, Barnes RD, Joffe J, Kahn D (1995). Emphysematous pyelonephritis in diabetic patients. Br J Urol.

[B10] Adbul-Halim H, Kehinde EO, Abdeen S (2005). Severe emphysematous pyelonephritis in diabetic patients: diagnosis and aspects of surgical management. Urol Int.

